# Anodal tDCS over the Motor Cortex on Prepared and Unprepared Responses in Young Adults

**DOI:** 10.1371/journal.pone.0124509

**Published:** 2015-05-01

**Authors:** Alexander C. Conley, Jodie Marquez, Mark W. Parsons, W. Ross Fulham, Andrew Heathcote, Frini Karayanidis

**Affiliations:** 1 Functional Neuroimaging Laboratory, School of Psychology, University of Newcastle, Newcastle, Australia; 2 Priority Research Centre for Translational Neuroscience and Mental Health, University of Newcastle, Newcastle, Australia; 3 Hunter Medical Research Institute, Newcastle, Australia; 4 School of Health Sciences, University of Newcastle, Newcastle, Australia; 5 School of Medicine and Public Health, University of Newcastle, Newcastle, Australia; The University of Queensland, AUSTRALIA

## Abstract

Anodal transcranial direct current stimulation (tDCS) over the primary motor cortex (M1) has been proposed as a possible therapeutic rehabilitation technique for motor impairment. However, despite extensive investigation into the effects of anodal tDCS on motor output, there is little information on how anodal tDCS affects response processes. In this study, we used a cued go/nogo task with both directional and non-directional cues to assess the effects of anodal tDCS over the dominant (left) primary motor cortex on prepared and unprepared motor responses. Three experiments explored whether the effectiveness of tDCS varied with timing between stimulation and test. Healthy, right-handed young adults participated in a double-blind randomised controlled design with crossover of anodal tDCS and sham stimulation. In Experiment 1, twenty-four healthy young adults received anodal tDCS over dominant M1 at least 40 mins before task performance. In Experiment 2, eight participants received anodal tDCS directly before task performance. In Experiment 3, twenty participants received anodal tDCS during task performance. In all three experiments, participants responded faster to directional compared to non-directional cues and with their right hand. However, anodal tDCS had no effect on go/nogo task performance at any stimulation – test interval. Bayesian analysis confirmed that anodal stimulation had no effect on response speed. We conclude that anodal tDCS over M1 does not improve response speed of prepared or unprepared responses of young adults in a go/nogo task.

## Introduction

There is a growing body of literature on the potential therapeutic effects of anodal transcranial direct current stimulation (tDCS) across a wide range of physical and mental pathologies. However, despite many reports of functional benefits in areas as diverse as motor recovery post-stroke and depressive symptomology, little is known about the mechanisms by which tDCS produces these improvements. In this study, we examined the effect of anodal tDCS over the motor cortex (M1) on prepared and unprepared motor responses in healthy young adults using a go/no-go paradigm.

Transcranial direct current stimulation (tDCS) is the generation of a weak electrical current through the neocortex via scalp electrodes [1, 2]. Sustained application of a mild electrical current has been shown to generate changes at the synaptic level that persist following cessation of stimulation [1]. The nature of these ‘after-effects’ depends on the polarity of stimulation. tDCS delivers subthreshold stimulation that affects neuronal firing rate by manipulating the balance of ions inside and outside the neural membrane. Cathodal stimulation produces hyperpolarization whereas anodal stimulation produces depolarization of the resting membrane potential [3]. Typically, studies compare a 5–30 minute period of active stimulation against a sham condition, which consists of a brief 30 second ramp-up of current followed by a rest period of the same duration as the active stimulation [2]. As sensory effects are generally limited to the early phase of stimulation, this sham stimulation condition tends to be indistinguishable from the active stimulation condition [2].

### Effects of tDCS on M1

Most studies examining the effects of anodal tDCS on motor processes have looked at one of two outcome measures: output of excitability or functional motor tasks. The effect of anodal tDCS over M1 on motor output of hand and leg muscles has been examined in young adults by measuring changes in the amplitude of the motor evoked potential (MEP) elicited by transcranial magnetic stimulation (TMS). Specifically, when applied to the motor cortex, TMS elicits a MEP that is recorded at the effector muscle using electromyography (EMG). Nitsche & Paulus [1] reported changes in TMS-elicited MEP amplitude both during and after the cessation of tDCS. Following five minutes of anodal tDCS, the amplitude of the MEP that was elicited by TMS was significantly greater than baseline and remained increased for a further five minutes. Longer stimulation periods produced more sustained changes to MEPs. For instance, thirteen minutes of anodal tDCS over the dominant (left) M1 increased TMS-elicited MEP amplitude at the right abductor digiti minimi muscle (ADM) for more than one hour [4]. Similar effects of anodal tDCS on motor excitability output have been shown at the tibialis anterior muscle (TA) in the leg [5]. The mechanisms behind these effects of anodal tDCS have been examined using pharmacological agents. Post-stimulation effects on MEPs were abolished following NMDA blockers, indicating that changes in excitability from tDCS may enhance a long-term potentiation (LTP)-like mechanism [6].

The effects of anodal tDCS on gross and fine motor skills have also been examined, especially as these have direct clinical implications. Gross motor tasks regularly used in clinical studies include the Jebsen Taylor Hand Function Test (JTT; [7]) and grip force measures. Following anodal tDCS over the dominant M1, young adults showed faster JTT completion [8] and increased isometric force [9] with the *non-dominant* hand compared to sham. This paradoxical improvement in non-dominant hand performance, in the absence of an effect on dominant hand performance, has been attributed to a ceiling effect as the dominant (right) hand of young adults is likely to be at peak excitability [10].

The effect of anodal tDCS on fine motor tasks has been assessed using accuracy of motor sequence completion. Anodal tDCS over the dominant M1 in right-handed adults produced more accurate responses to learned number sequences [11–13]. Bihemispheric anodal tDCS increased response accuracy to randomly presented letter sequences for both left and right hands [14]. This improved response accuracy following anodal tDCS over M1 in young adults has been attributed to increased efficiency of motor pathways. However, as response accuracy only captures the end point of a series of cognitive and motor processes, it is not a direct measure of motor pathway efficiency. Moreover, motor sequence tasks do not differentiate between cognitive and motor processes contributing to final motor output.

### Present Study

In this study, we examined the effects of anodal tDCS over the motor cortex on behavioural performance. As the motor cortex is directly involved in the generation of movement, facilitation as a result of anodal tDCS should lead to improved performance, and especially faster reaction time (RT). We used a cued go/nogo paradigm in order to examine whether anodal tDCS over the left M1 in healthy young adults would differentially affect performance of prepared and unprepared motor responses. A double-blind randomised controlled design was used, with anodal tDCS and sham sessions separated by a three-week washout period. Based on previous findings of increased motor excitability and improved motor sequence and functional motor task performance following stimulation (see [15] for a review), we hypothesised that anodal tDCS would improve performance (i.e. increase response speed and reduce error rate) compared to the sham condition.

## Experiment 1

In Experiment 1, active tDCS stimulation of the dominant (left) motor cortex was expected to improve performance compared to sham stimulation, and the effect was expected to be greater for the right (contralateral) hand. However, given earlier findings in young adults, and the suggestion that there may be a ceiling effect for dominant hand performance, this pattern may be reversed. Moreover, if tDCS improves performance by acting on motor preparation processes, we expected the effect to be greater for directional than non-directional cue conditions.

### Methods

#### Participants

Twenty-four participants (9 males, mean age ± standard error: 21.3 ± 2.5yrs) were recruited from a 1^st^ year research volunteer group at the University of Newcastle. Participants were screened for suitability for direct current stimulation (i.e., excluding epilepsy, major heart condition, neurological disease, metal implants). All participants were right handed as measured by the Edinburgh Handedness Inventory [16]. Order of active and sham stimulation conditions was randomised between subjects. Twelve participants (4 males, 20.8 ± 0.5yrs) received sham in the first session and 12 participants (5 males, 21.8 ± 0.9yrs) received active tDCS in the first session.

#### Ethics statement

This study protocol was approved by the University of Newcastle’s Human Research Ethics Committee (H-2013-0115), and complied with the Declaration of Helsinki. All participants gave written informed consent before beginning participation in this study. This consent form was approved by the University of Newcastle’s Human Research Ethics Committee.

#### Transcranial direct current stimulation settings

Anodal tDCS stimulation was delivered by a battery-driven constant-current stimulator (neuroConn GmbH, Germany) and involved the application of a 1mA current for 20 minutes using two rubber electrodes (35cm^2^) soaked in saline. The current density of the electrodes was 28.6μA/cm^2^. The anode was placed over the left primary motor cortex (M1), while the cathode was placed over the supraorbital region of the contralateral hemisphere. The location of the hand area over M1 was determined as the C3 electrode according to the international 10/20 system, as used in [17]. This montage has been shown to be effective at increasing the excitability of the dominant motor cortex [1, 4].

Sham stimulation involved the application of a 1mA current for 40s (10s ramp up and 30s stimulation) followed by 20min delay to match the duration of the active stimulation session. Both experimenter and participant were blind to the order of stimulation. An experimenter who was not involved in testing determined a pseudo-random sequence of active/sham stimulation orders, so as to have equivalent numbers in each order. Participants were sequentially allocated to one of these. The two sessions were scheduled at least three weeks apart to avoid any carry over effects of tDCS stimulation.

#### Cued go/nogo paradigm

Each trial began with a fixation cross (500ms). This was replaced by a cue (S1) which validly predicted the onset of the target (S2) after a fixed cue-target interval (CTI = 1500ms). The target remained visible for 1000ms during which interval a response was emitted. The interval between target onset and the onset of the next cue was jittered with a mean of 2000ms (random sequence, 1500–2500ms).

Directional and non-directional cues were presented in different blocks that were delivered in a randomised order. On non-directional cue blocks, the cue consisted of two black arrowheads that pointed in different directions (i.e., <>) and validly predicted the timing of target onset but not the response required. The target was two green directional arrowheads (i.e., <<, >>) that specified whether to make a left-hand or a right-hand response with the respective index-finger using buttons attached to the arm rests. During the CTI, participants could prepare to process the target, but were not aware whether they would need to prepare a left-hand or a right-hand response. On directional cue blocks, the cue was two black arrowheads that validly predicted the required response. The targets were the same as for non-directional cues. On 30% of the trials, the target was a ‘no-go’ stimulus (i.e., a red X) indicating that a response must be withheld. So, on these informative cue blocks, participants could use the cues to prepare a left-hand or a right-hand response, but had to await target onset to check whether the response must be withheld. Participants completed five blocks of 80 trials: two with non-directional and three with directional cues Prior to testing on each session, participants completed one practice block of thirty trials on each cue type.

#### Procedure

In each session, participants received a 20 minute period of either sham or anodal stimulation. This was followed by completion of the Grooved Pegboard Test [18], the Digit Span test [19], the Trail Making Test [20] and two practice blocks of the cued go/nogo paradigm. These tests assessed whether anodal tDCS over M1 affected fine motor skills, working memory and set-shifting. Participants were then prepared for EEG recording and completed the cued go/nogo paradigm. Results of the EEG results will be reported separately. The go/nogo task began approximately 40min after termination of stimulation. At the completion of each session, participants were given a short questionnaire assessing their comfort during the tDCS intervention and whether they believed they had received active or sham stimulation in that session.

#### Statistical analysis

Response speed on go trials was analysed using a four-way mixed-design GLM with Order (sham first, active first) as a between-groups condition and three repeated measures conditions: Stimulation (active, sham), Cue (directional, non-directional) and Response Hand (left, right). Standard error of the mean was calculated using the procedure developed by Morey [21]. Data from Digit Span, Trail Making and Grooved Pegboard tests were analysed using a three-way mixed-design GLM with Order, Stimulation and Digit Sequence (forward, backward), Trail Type (A,B), or Response Hand (left, right), respectively.

### Results

Some participants reported experiencing mild discomfort during the initial phases of stimulation in both active and sham sessions. The most common sensation was a mild itching or prickling sensation under the electrodes, but no participant requested to discontinue testing. Chi-square analysis showed that participants did not accurately predict whether they had received active or sham (*x* ^*2*^ (1) = 1.5, *p* = 0.22).

Table 1 show means for the Digit Span, Trail Making and Grooved Pegboard tasks. On the Digit Span test, there was no effect of stimulation or interaction between stimulation and other factors. On the Trail Making Test, performance was faster on Trails A than Trails B (*F* (1, 21) = 66.45, *p*<0.001). On the Grooved Pegboard test, responding was faster with the right than the left hand (*F* (1, 21) = 14.5, *p* = 0.001). Both tests showed no main effect of stimulation (both *F* (1, 21) < 1). Additionally, they both produced a significant interaction between stimulation and order (*F* (1, 21) = 7.58, *p* = 0.012; *F* (1, 21) = 21.63, *p* <0.001), indicating that responding was faster on the second session, irrespective of stimulation condition.

**Table 1:** Results for Digit Span, Trail Making and Pegboard tests in Experiment 1.

Test	Active Mean	Sham Mean	t-Statistic	P
**Digit Span**	11.1	11.2	-0.448	0.66
Trail Making*	28.28	31.99	-0.693	0.5
**Pegboard**	62.93	62.97	-0.034	0.97

*Difference score between Trails A and Trails B

**Fig 1:**
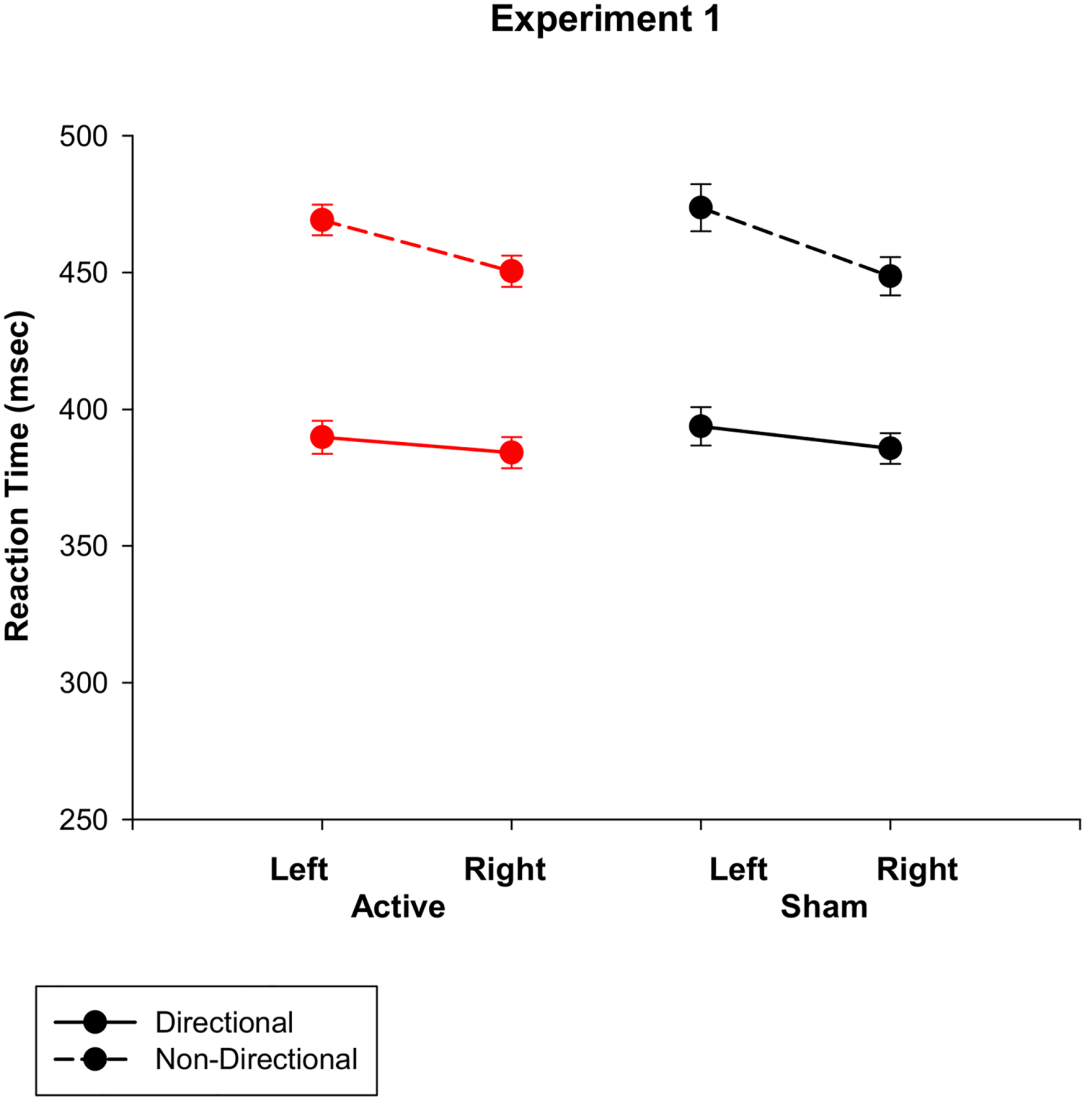
Reaction time for directional and non-directional cue conditions under active vs. sham stimulation for Experiment 1.

Fig 1 shows reaction times for directional and non-directional cue conditions under active and sham stimulation. As error rate on go trials (2.23%) and false alarm rate for no-go trials (2.03%) were very low, they are not statistically analysed. Response time was faster for directional than for non-directional cues (*F* (1, 22) = 132.6, *p*<0.001; 388 vs 460 ms) and for right than left hand responses (*F* (1, 22) = 22.6, *p*<0.001). This right hand advantage was larger for non-directional cues (Cue*Hand: *F* (1, 22) = 9.4, *p* = 0.006). As shown in Fig 1, stimulation had no effect on RT (423 vs 425 ms, *F* <1), and did not interact with other factors (Stimulation*Cue: *F*<1; Stimulation*Hand: *F* (1, 22) = 1.76, *p* = 0.2). There was also no main effect of order (F<1) or interaction between order and other factors (Stimulation*Order: *F*<1; Cue*Order: *F*<1; Hand*Order: *F* (1, 22) = 1.75, *p* = 0.2).

### Discussion

On the cued go/nogo task, participants showed the expected advantage for directional versus non-directional cues and for right vs. left hand responses. Error and false alarm rates were low for both cue conditions. These results confirm that participants were attending to the task, using cues to prepare their response and waiting for the target before responding on directional cue blocks. Contrary to our predictions, there was no evidence for a beneficial effect of anodal tDCS over M1 on response speed for either directional or non-directional cues (Fig 1). We conclude that response preparation and response selection as assessed by a cued go/nogo task are not affected by anodal tDCS over dominant M1 in young adults.

The finding that, in young adults, dominant hand performance is not enhanced by anodal tDCS over the dominant M1is consistent with the JTT findings by Boggio et al. [8]. However, Boggio et al. [8] did find improvement of non-dominant hand performance, and concluded that, in healthy young adults, stimulation improves JTT performance with the less efficient non-dominant. The current data do not support this conclusion, as tDCS did not improve responding with either the dominant or the non-dominant hand. Indeed, the marginal interaction between stimulation condition and response hand (p = 0.13) was driven by participants being slightly *slower* with their dominant hand following stimulation compared to sham.

The absence of a significant effect is unlikely to result from our choice of stimulation parameters, as they were selected to match optimal parameters defined by Nitsche & Paulus [1]. However, the long delay between the cessation of stimulation and the start of the cued go/nogo task may have contributed to the absence of a stimulation effect. In this experiment, the go/nogo task did not commence until approximately 40 minutes after stimulation, because participants first completed the fine motor and working memory tasks, practiced the experimental task and were set up for EEG recording. Therefore it is possible that the effects of stimulation may have been attenuated before the onset of testing. Note, however, that this is unlikely, as performance on the grooved pegboard task, which requires fine motor control and was performed directly following stimulation, was also not affected by anodal tDCS. Nevertheless, in order to examine whether the delay between stimulation and test may be responsible for the absence of an effect of anodal tDCS on response speed on the cued go/nogo task, we completed two further experiments that manipulated the delay between stimulation and testing.

## Experiment 2

In Experiment 2, the test session commenced immediately after stimulation. To minimise the interval between stimulation and task onset, we did not include the Grooved Pegboard, Digit Span and Trail Making tasks, and recorded only behavioural data on the cued go/nogo paradigm.

### Methods

Eight right-handed participants (3 male, 22.3±3.4yrs) completed the cued go/nogo directly after active and sham stimulation conditions. Except as specified, all task, stimulation and analyses parameters were identical to those used in Experiment 1. Participants completed task practice before receiving the stimulation intervention, to reduce any delay between stimulation and test.

### Results and Discussion

**Fig 2:**
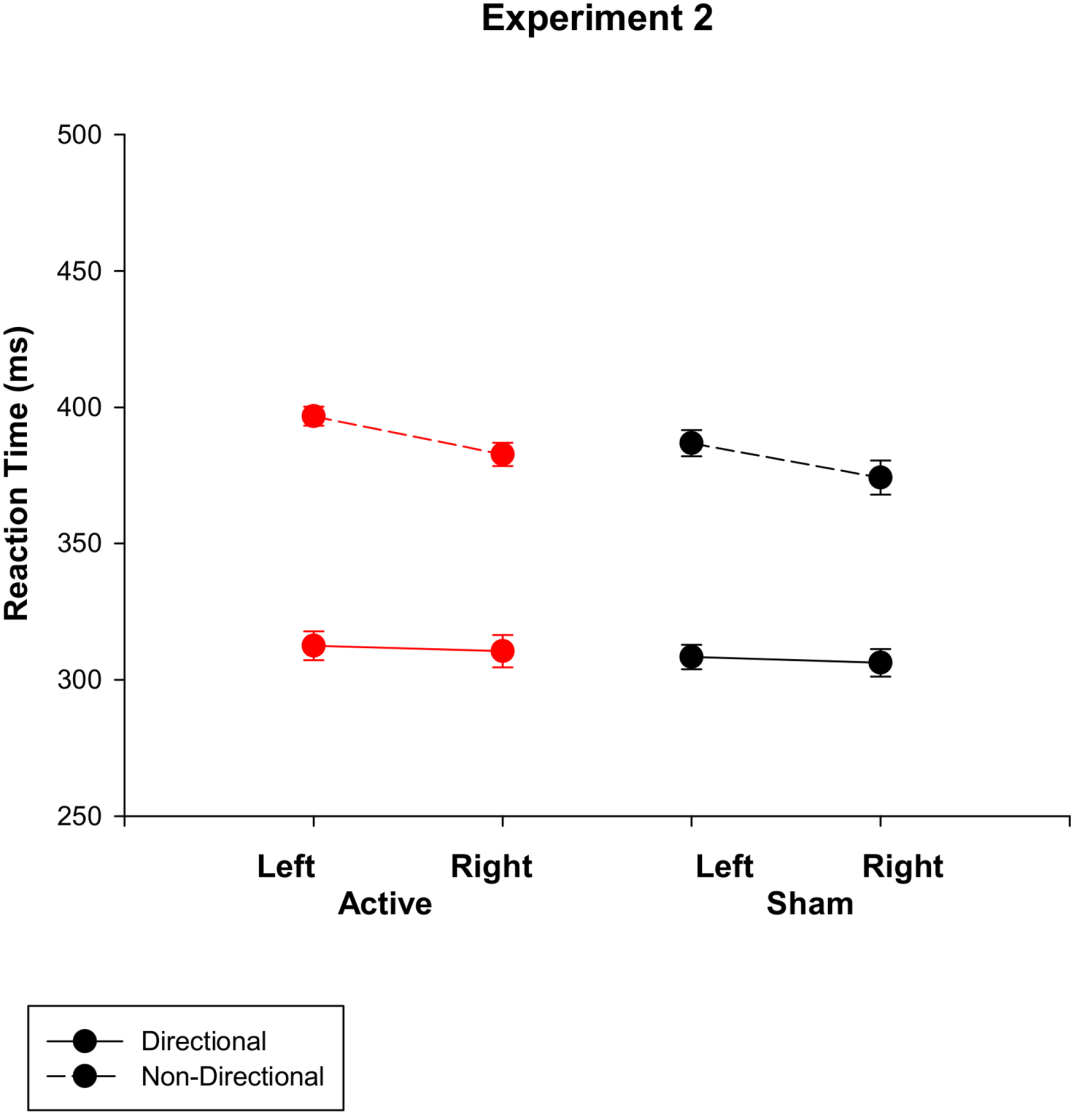
Reaction time for directional and non-directional cue conditions under active vs. sham stimulation for Experiment 2.

Participant reported similar physiological sensations as in Experiment 1. Error and false alarm rates were again very low (2.2% and 3.2% respectively) and were not statistically analysed. Fig 2 shows that mean RT was faster for directional compared to non-directional cues (*F* (1, 6) = 193, *p*<0.001; 309 vs 385 ms) and for right than left hands (*F* (1, 6) = 8.4, *p* = 0.027). This right hand advantage was larger on blocks with non-directional compared to directional cues (*F* (1, 6) = 14.1, *p* = 0.009). Despite there being no delay between stimulation and task onset, there was again no effect of anodal tDCS on RT (*F* (1, 6) = 1.18, *p* = 0.32), or any interaction between stimulation and any other factor (*F*<1). While the sample used in this study was smaller than that in Experiment 1, the pattern of results was highly consistent. Thus, Experiment 2 supports the conclusion that anodal tDCS over the motor cortex has no effect on response speed on a cued go/nogo task.

## Experiment 3

While most studies assess the effects of tDCS on subsequent behaviour, others use online stimulation i.e., assess performance during the course of stimulation [11, 22, 23]. As Experiments 1 and 2 showed no effects of tDCS on the cued go/nogo task when it was completed after stimulation, in Experiment 3, we assessed whether the expected improvement of RT on prepared and unprepared motor responses may be induced by online tDCS.

### Methods

Twenty right-handed participants (7 males, 21.2±2.9yrs) completed active and sham stimulation conditions in a pseudorandom sequence. All task parameters were identical to Experiment 2, with the exception that the cued go/nogo task commenced immediately after the onset of stimulation.

### Results

**Fig 3:**
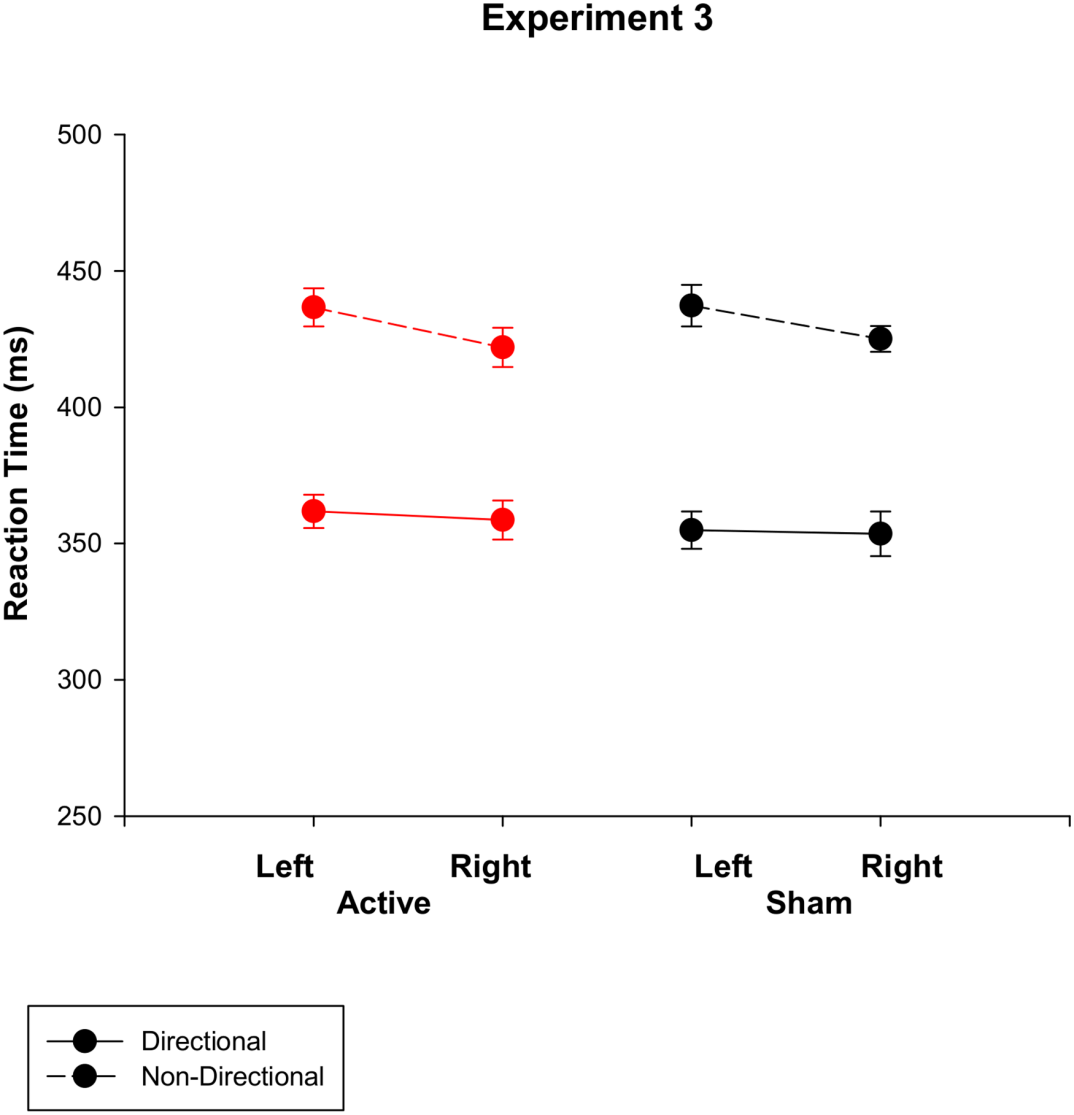
Reaction time for directional and non-directional cue conditions under active vs. sham stimulation for Experiment 3.

As in previous experiments, participants reported mild itching sensations. Error rates were again very low (2.3% incorrect, 2.1% false alarms). As shown in Fig 3, RT was faster for directional than non-directional cues (*F* (1, 18) = 109.7, *p*<0.001; 357 vs 430ms), and the right hand RT advantage was greater for non-directional cues (*F* (1, 18) = 8.175, *p* = 0.01). Once again, there was no main effect of anodal tDCS (*F* (1, 18) = 0.6, *p* = 0.8).

There was, however, a main effect of stimulation order, as RT was faster for participants who received active stimulation first than participants who received active second (*F* (1, 18) = 9.57, *p* = 0.006; 364 vs 423 ms). There was also a three-way interaction between stimulation, response hand and stimulation order (*F* (1, 18) = 6.99, *p* = 0.017). This effect emerged because left-hand responses following active tDCS were slower for participants who received stimulation second compared to those who received stimulation first.

### Discussion

As in Experiments 1 and 2, participants responded faster to directional than non-directional cues and showed a right hand advantage, especially for non-directional cues. Despite receiving active tDCS simultaneously with task performance, again responding was not faster under active than sham stimulation conditions. Therefore, across the three experiments, we show a highly consistent finding that, in young adults, anodal tDCS over left motor cortex does not improve response speed with either the dominant or the non-dominant hand. Importantly, Experiment 3 shows that this null effect cannot be accounted for by the length of the delay between stimulation and test.

Again, however, our result is not consistent with some previous studies. For example, Nitsche et al. [11] showed that the improvement in response speed was greater with online than offline application of anodal tDCS over M1. They concluded that online application of anodal tDCS produces additive enhancement of response processes. However, this is not a consistent finding. For example, Miyaguchi et al. [24] found worse performance following online anodal tDCS compared to offline tDCS.

## Bayesian Analyses across the Three Experiments

All three behavioural experiments failed to reject the null hypothesis that anodal tDCS has no effect on motor performance in a cued go/nogo task. However, in frequentist statistics, failure to reject the null hypothesis does not allow us to conclusively affirm that anodal tDCS has *no* effect on motor performance on this task. In order to test the strength of this null hypothesis, we took a Bayesian approach using the default-prior method for linear models [25] to repeat the factorial ANOVA analyses, including a between-subjects factor of timing of stimulation (delayed, immediate, simultaneous). This analysis produces Bayes factors, which equal the ratio of the evidence for one vs. another model, assuming the models are equally likely a-priori, and quantify the factor by which the data should change prior beliefs about the relative merits of each model. A Bayes factor greater than 10 is often considered as providing strong evidence for a model [26, 27]. We performed the analysis using the *BayesFactor* package in R [28] calculating Bayes factors for all hierarchical ANOVA models (i.e., higher order terms are only included with their constituents). We report Bayes factors assuming the default setting for the fixed-effect prior (r = 0.5).

The analysis showed that the best model included a main effect of cue (directional vs. non-directional), hand (left vs. right), stimulation order (active first vs. sham first), and timing (delayed, immediate, simultaneous), as well as an interaction between order and timing of stimulation The between group effect of timing emerged because participants in Experiment 2 had overall faster RT than the other two groups, especially when receiving active stimulation in the 1^st^ session. The absence of a main effect of stimulation or an interaction between stimulation and timing shows no behavioural benefit of anodal tDCS, regardless of when it was received in respect to the cued go/nogo task. The Bayes factor for this model relative to the best model that included one or more effects of stimulation (a single main effect) was 11.1. In other words, a null effect of stimulation is favoured by a factor of around eleven, indicating strong evidence that anodal tDCS has no effect on RT in young adults. Therefore, Bayesian analysis of data pooled across the three experiments supported the hypothesis that anodal tDCS over the dominant motor cortex is statistically unlikely to improve response speed in young adults.

## General Discussion

In this study, we investigated the effects of anodal tDCS on motor responding in a cued go/nogo task in healthy young adults. All participants showed good task performance with very low error and false alarm rates. In addition, as expected, they responded faster for directional (prepared) than non-directional (unprepared) cues and with their right (dominant) than their left hand. Anodal tDCS was applied over the dominant (right) hemisphere and order of stimulation (active or sham first) was randomised, with both participant and experimenter being blind to the stimulation condition. A long 3-week washout period was used to avoid any contamination between stimulation and sham. We chose stimulation parameters previously shown to be effective in facilitating motor output and gross motor performance [1].

Contrary to our original hypothesis, anodal tDCS of the dominant motor cortex did not significantly improve performance relative to sham stimulation on either prepared (directional) or unprepared (non-directional) responses performed with either the contralateral (stimulated) or the ipsilateral (non-stimulated hand). Given the unexpected absence of a significant stimulation effect in Experiment 1, we asked whether the relatively long delay (40 min) between stimulation and the onset of the cued go/nogo task may have resulted in an attenuation of the effect of stimulation. To test the possibility that the stimulation-test delay was responsible for the absence of a stimulation effect, we conducted two more experiments in which stimulation was applied immediately before task onset (Experiment 2) or during task completion (Experiment 3). Both experiments produced results consistent with Experiment 1; although participants performed the task well, performance was not enhanced by anodal tDCS over the dominant M1. Bayesian analysis confirmed the strength of the null hypothesis for stimulation. Across the three experiments, the most appropriate model did not include a positive effect of anodal tDCS on performance. Indeed, the strongest model that included stimulation was eleven times less powerful than the strongest model without stimulation. We therefore conclude that anodal tDCS over the motor cortex has no effect on speed of responding on a cued go/nogo task in healthy young adults.

This conclusion is at odds with prior evidence of enhanced TMS-induced MEP following anodal stimulation of dominant M1 in young adults [1] and improved motor performance on functional tasks of fine and gross motor skills, such as the JTT [8]. These early findings have set the framework regarding possible beneficial effects of anodal tDCS over the motor cortex in clinical rehabilitation. However, more recent research using anodal tDCS over M1 has not produced consistently positive findings. For instance, while a number of studies report that anodal tDCS over M1 enhances accuracy and response speed on finger-tapping sequence tasks [12–14], Leite et al. [29] found no effect of anodal tDCS over M1 on response accuracy or RT using a motor sequence task.

Inconsistent effects have also been reported in studies that examine the effects of anodal tDCS over M1 on response inhibition in young adults using the stop-signal task (SST). Kwon & Kwon [23] found that anodal tDCS over M1 improved response inhibition (i.e., reduced stop-signal reaction time, SSRT), while Hsu et al. [22] reported no effect on SSRT following stimulation over either M1 or pre-SMA. Effects of anodal tDCS on response accuracy in the stop-signal task have also been inconsistent. Hsu et al. [22] showed a reduction in false alarm rate following pre-SMA stimulation, whereas Hadyuk-Costa, Drummond & Carlsen [30] showed increased false alarm rate after anodal tDCS over M1. Anodal tDCS over M1 in healthy older adults also showed no beneficial effects on a choice RT task [31]. Pellicciari, Brignani & Miniussi [32] found that anodal tDCS over M1 did not improve response speed on a speeded detection task, over and above the effect of sham.

These discrepancies may be partly related to differences in task properties. Bortoletto et al. [33] found that simultaneous application of anodal tDCS over the motor cortex reduced performance compared to sham on a fast motor task, but not a slow motor task. Indeed, in a recent review, Miniussi, Harris & Ruzzoli [34] concluded that, in novel tasks, rather than facilitating processing efficiency, anodal tDCS may produce more cortical noise and interfere with task processing. This could explain the difference between findings with sequenced tapping tasks [12–14] and tasks with more complex demands.

Alternatively, the absence of an effect of tDCS on response time in the cued go/nogo task may be related to the stimulation parameters used. The specific montage used here (i.e., active electrode over left motor region and reference over right supraorbital region) was selected as it has been shown to stimulate the motor cortex [11]. However, given the poor focal nature of tDCS, it is likely that stimulation spread across a greater area on the surface of the neocortex [35]. Moreover, the placement of the reference electrode over the contralateral supraorbital region may have attenuated the effect of the anode by decreasing activation of the right orbitofrontal area. Nevertheless, the fact that these stimulation parameters have been previously shown to positively affect motor task performance indicates that this is unlikely to account for the lack of stimulation effects in this study.

An alternative explanation for the lack of a stimulation effect may be that our healthy young adults were performing at peak and had no room to improve response speed. This is supported by the low error rate. However, participants showed clear response time differences between prepared and unprepared responses, as well as between left and right hands. Even if prepared right hand responses were at ceiling, stimulation could have improved unprepared and/or left hand responses. Moreover, positive effects of anodal tDCS have been reported in young adults even with simple motor tasks, such as with the JTT [8] or force measurement [9].

In conclusion, the present study shows that anodal tDCS over the dominant motor cortex in young people did not improve speed of responding to either directional or non-directional cues in a cued go/nogo task. This effect was replicated in three experiments which varied the timing between stimulation and test. Recently, Pellicciari, Brignani and Miniussi [32] suggested that anodal tDCS may modulate the efficiency of cortico-motor pathways, instead of eliciting direct excitation. It is therefore possible that, although there is no effect on overt behavioural outcome, tDCS may produce subtle differences in cognitive and motor processes that contribute to response selection and activation. Event-related potentials (ERP) allow the measurement of electrophysiological components associated with cognitive and motor processes at different temporal windows and may help address this question.
